# Application of phototherapeutic-based nanoparticles in colorectal cancer

**DOI:** 10.7150/ijbs.58773

**Published:** 2021-04-02

**Authors:** Jiaxin Yan, Chunli Wang, Xiaomei Jiang, Yiqu Wei, Qun Wang, Kunli Cui, Xiao Xu, Feng Wang, Lei Zhang

**Affiliations:** 1 Bioinformatics Center, School of Basic Medical Sciences, Henan University, Kaifeng 475004, China.; 2 School of Basic Medical Sciences, Henan University, Kaifeng 475004, China.; 3 School of Pharmacy, Henan University, Kaifeng Kaifeng 475004, China.; 4 Guangming Substation of Shenzhen Ecological Environment Monitoring Station, Shenzhen 518107, P. R. China.

**Keywords:** colorectal cancer, photosensitizer, photothermal therapy, photodynamic therapy, nanoparticle.

## Abstract

Colorectal cancer (CRC) is the third most commonly diagnosed malignancy and the second leading cause of cancer death, which accounts for approximately 10% of all new cancer cases worldwide. Surgery is the main method for treatment of early-stage CRC. However, it is not effective for most metastatic tumors, and new treatment and diagnosis strategies need to be developed. Photosensitizers (PSs) play an important role in the treatment of CRC. Phototherapy also has a broad prospect in the treatment of CRC because of its low invasiveness and low toxicity. However, most PSs are associated with limitations including poor solubility, poor selectivity and high toxicity. The application of nanomaterials in PSs has added many advantages, including increased solubility, bioavailability, targeting, stability and low toxicity. In this review, based on phototherapy, we discuss the characteristics and development progress of PSs, the targeting of PSs at organ, cell and molecular levels, and the current methods of optimizing PSs, especially the application of nanoparticles as carriers in CRC. We introduce the photosensitizer (PS) targeting process in photodynamic therapy (PDT), the damage mechanism of PDT, and the application of classic PS in CRC. The action process and damage mechanism of photothermal therapy (PTT) and the types of ablation agents. In addition, we present the imaging examination and the application of PDT / PTT in tumor, including (fluorescence imaging, photoacoustic imaging, nuclear magnetic resonance imaging, nuclear imaging) to provide the basis for the early diagnosis of CRC. Notably, single phototherapy has several limitations *in vivo*, especially for deep tumors. Here, we discuss the advantages of the combination therapy of PDT and PTT compared with the single therapy. At the same time, this review summarizes the clinical application of PS in CRC. Although a variety of nanomaterials are in the research and development stage, few of them are actually on the market, they will show great advantages in the treatment of CRC in the near future.

## Introduction

CRC is the world's third-largest cancer in men (10.9% of the total incidence), the second-largest in women (9.5% of the total incidence), and the second leading cause of death due to oncological causes 1. CRC is the major burden to human health, due to late diagnosis and ineffective treatment approaches. Several methods are used for treatment of CRC, such as chemotherapy, surgical resection and other traditional treatments. Adriamycin is one of the most effective anti-tumor drugs used for treatment of a variety of tumors 2. In addition, some compounds can inhibit the proliferation of CRC by reducing the content of cysteine and glutathione, thereby inducing the accumulation of reactive oxygen species (ROS) and ferroptosis 3. Currently, surgical excision is the conventional treatment approach for CRC 4, surgery method is highly effective for early stage CRC. However, CRC is poorly diagnosed at early stages and early symptoms are easily confused with symptoms for other diseases. Therefore, most of CRC patients are diagnosed during advanced or metastatic stages 5. Notably, tumors in most advanced CRC patients cannot be resected and often resistant to conventional chemotherapy. Most of CRC patients relapse after surgery and adjuvant chemotherapy during early disease stages 6, 7. In addition, chemotherapeutic drugs are associated with toxicity to healthy tissues due to poor specificity 8. Furthermore, these drugs have side effects, which are associated with poor specificity 9. Therefore, studies should explore novel and effective approaches for CRC treatment. More and more researches are devoted to the diagnosis and treatment of CRC at the cellular, subcellular and molecular levels. Recently, treatment of CRC using phototherapy was reported. Phototherapy is as old as the advent of human medicine. Application of phototherapy includes killing of harmful microorganisms by sun exposure and ablation of diseased tissue using highly focused lasers 9. Phototherapy involves interaction between light, PS and oxygen, and PS is the key to the treatment of CRC with phototherapy 8. PDT and PTT are the main phototherapy approaches, and this article will focus on these methods.

PSs and appropriate illumination are the necessary conditions for PDT to exert tumor inhibitory effect. PDT effect is based on the concentration difference of PSs accumulated between tumor tissue and normal tissue. Irradiation using a certain wavelength of laser, changes PS energy from the ground state to a singlet excited state. The singlet excited state can be maintained for a long time through high oxidation. In the process of transition to the ground state, a large amount of singlet oxygen (^1^O_2_) is accumulated in tumor tissue to induce apoptosis of tumor cells through what is usually thought of as Type II reaction (In general, upon excitation, the PS is pumped from the ground singlet state to an excited singlet state, then transformed into a relatively long-lived triplet state by intersystem crossing (ISC). Then it occurs energy transfer between the triplet state of the PS and an adjacent oxygen molecule, forming singlet oxygen 10-12.). In addition, energy conversion transfers electrons to biological molecules, water, oxygen and other substances, resulting in gradual accumulation of reactive molecules species in the tumor site. Although PDT is at the experimental stage, current findings show that it is effective for diagnosis and treatment of CRC 13.

The principle behind PTT is that after accumulation of PS in the tumor site, the energy of the specific wavelength laser is converted into heat energy, causing the thermal ablation of tumor cells. Compared with the traditional treatment, PTT has obvious advantages in high uniqueness, low invasiveness, reducing normal tissue toxicity and high drug metabolism. Notably, PTT is an effective treatment approach option for CRC 14 like PDT.

PDT and PTT are modern cancer treatment methods, and their basis of action is to act on the local or systemic photosensitive compounds, known as PSs. Photosensitive molecules absorb appropriate wavelength resulting in activation of these molecules, causing cell death ultimately 15. The characteristics of ideal PSs including: 1) high chemical purity; 2) relative stability at room temperature; 3) absorbance between 600 and 1000 nm, and photosensitive effect can only be produced at specific wavelength; 4) low toxicity to other tissues and cells, and easy dissolution in human tissues; 5) high selectivity to tumor tissues; and 6) cheap, simple synthesis and easy to obtain.

The selection and application of PS is basis of light therapy, studies on PS experienced a long process, and currently only a handful of PS to get official approval. Discovery of first-generation PSs was mainly motivated by a study by Munich ascar Raab on acridine dye. Protozoa treated with dye and subjected to radiation, resulting in oxygen consumption and toxic effect, eventually died, a phenomenon known as "photodynamic effect" 16. A water-soluble porphyrin mixture known as “hematoporphyrin derivative” (HPD) was discovered in 1970s as the first-generation PSs 15. However, PSs have low chemical purity and poor tissue permeability. Second-generation PSs include HPDs and some synthetic PSs, such as 5-aminolevulinic acid hydrochloride (5-ALA), benzoporphyrin derivatives, and phthalocyanine 17, 18. Compared with first-generation, second-generation PSs have many advantages, such as fewer side effects, higher chemical purity, higher ^1^O_2_ generation rate, and stronger permeability to deep tissues. However, second-generation PSs still have the limitation of poor water solubility 15. Development of third-generation PSs is aimed at achieving high affinity for tumor tissues to reduce toxicity on surrounding healthy tissues 15. Studies have explored several methods to improve the selectivity of drugs, such as using tumor surface markers to combine PS with low-density lipoprotein. Recently, nanotechnology has developed rapidly, and combination and application of surface PSs and nanoparticles improves efficiency of PDT and PTT approaches. PSs can be transformed into nanoparticle form to further improve application and the effectiveness against tumor cells or animal models.

Nanoparticles are used as drug carriers for photosensitive molecules in optical therapy, ensuring new functions and improving their effectiveness 19. For example; molecules with greater tissue penetration, can be used for PS delivery in PDT, whereas molecules with stronger light absorption, can be used as enhancers in PTT 20. Several nanoparticles for application in phototherapy have been developed and are currently used in clinical research. For instance: 1) Inorganic nanoparticles used in phototherapy include metal nanoparticles (including gold nanorods 21, gold nanocapsules, gold nanocages 22, and gold nanostars 23) whereas, carbon-based nanoparticles include fullerenes, carbon nanotubes, and graphene. In addition, other inorganic nanoparticles are used. 2) Organic nanoparticles for phototherapy include near-infrared (NIR) dyes, including indocyanine green (ICG) 24( a drug approved by Food and Drug Administration (FDA) for clinical use), conjugated polymer nanoparticles, and other organic nanomaterials, including phospholipid coated porphyrin. 3) Upconversion nanoparticles have stronger tissue penetration ability compared with the previously mentioned materials 25.

In this review, we introduce the development of PS, the main methods for improvement of PS, main PSs used in PDT/PTT and advances in clinical development and selectivity of PSs. In addition, we summarize the application of PDT and PTT in CRC treatment and explore their mechanisms of action. Further, we explore the effects of PDT and PTT alone and in coordination and their respective advantages and disadvantages. Moreover, we review research and development of several nanotechnologies and nanoparticles relevant to phototherapy for CRC treatment. Furthermore, application of PDT, PTT and deep tissue imaging in tumor tissues and the application of phototherapy combined with other therapies for CRC treatment are introduced.

## PSs and improved methods

### The process of PSs induced phototherapy and the characteristics of PSs

Light trigger therapy directly destroys cancer cells, and induces post-treatment effects. It improves efficacy of combined therapy by enhancing intratumoral penetration and entry of nanoparticles into cells, accelerating drug release and improving immune response to cancer cells^26^. PDT kills cancer cells and improves survival and quality of life in cancer patients. Several new approaches have been developed to improve efficacy of PDT^27^. PS is irradiated to produce ROS or monomorphic oxygen that acts on cancer cells and causes irreversible damage to cancer cells through photo action 28. Targets for photo action include epidermal growth factor receptor, cholesterol and low-density lipoprotein, estrogen receptor, nucleus and DNA, folic acid receptor, cholecystokinin receptor, lectin carbohydrate receptor and tumor-specific antibody 29. Halogen lamps, arc lamps, lasers and light-emitting diodes are used to activate PS, however they are all visible light with high tissue absorption, leading to low tissue penetration thus have low treatment efficacy. NIR region wavelength ranges from 600 to 1000 nm and can minimize tissue attenuation to maximize effectiveness by ensuring tissue penetration 9. Long wavelength light activates PS and kills cancer cells concurrently, however, it causes irreversible damage to normal cells. Therefore, excitation wavelengths of PS used in treatment of CRC is mainly concentrated in the NIR region. PSs produce heat just like light action to kill cancer cells under different wavelengths of light in a process-known as photothermal action. In addition, when tumor is heated to 42°C by photothermal action, the cancer cells are more susceptible to other treatments 26. Photosensitive therapy has unique advantages such as spatiotemporal selectivity, high efficiency, no drug resistance and non-invasiveness which are not present in conventional approaches. Excitation wavelength of PS in the NIR has the advantages of NIR light activation, low invasive treatment, non-specific activation, high temporal and spatial accuracy, and real-time dose adjustment. PSs can be grouped into various types based on their carriers, including inorganic PSs, organic PSs and metal PSs 26. Although PSs can kill cancer cells, preclinical and clinical studies report a few shortcomings, which hinder their development. Therefore, PSs should be modified to reduce its limitations and improve effectiveness.

### Improvement of PS

#### Increasing penetration depth

Different wavelengths of light have different ability to penetrate tissue. Longer wavelength of light show, stronger ability to penetrate tissue compared with short wavelength light. For instance, the activation wavelength of photofrin is 630 nm, however, its penetration depth is only about 0.5 cm, therefore, it is not ideal for large or deep tumors. On the other hand, with wavelength of the light source approximately 800 nm, results in tissue penetration depth of 1 cm. However, short-wavelength light sources have higher ^1^O_2_ yield and shorter irradiation time compared with long-wavelength sources 30. One of the solutions to improve the penetration of deep tissue lesions is to use the long wave near-infrared (NIR) light in the range of 600 ~ 1000 nm, known as the "NIR window" or "optical window" 31. NIR light is preferred 32. Therefore, current studies are exploring the best irradiation wavelength with high ^1^O_2_ yield. Studies report that femtosecond lasers are used to drill holes in tumor tissue in advance to enhance tissue permeability of PS, thus improving its killing depth. However, studies have not explored the effect of this method on spreading of tumors 30.

#### Enhanced targeting

Tumor cells require higher glucose levels compared with normal cells (a phenomenon known as the Warburg effect 33) and this effect can be extended to a variety of other carbohydrates, including mannitose and galactose 34. Therefore, recent studies have exported conjugation of PSs with sugars to increase selective recognition or uptake by cancer cells. Nanomedicine and nanoparticle therapy have several advantages compared with traditional therapies, however their specificity is low, and these methods have poor pharmacokinetics. Nanoparticles have attracted extensive research attention due to the variety of potential modification 35. Structure of nanoparticles can be used as a multifunctional assembly platform 36-38, enabling combination of chemotherapy drugs and cancer imaging agents 39. The structure ensures high solubility and colloidal properties, which can be used in complex environments (such as blood and tissue)^28^. Gold nanoparticles, for example, have good biocompatibility and can accumulate passively within tumors by enhancing permeability and retention. Moreover, gold nanoparticles are efficient at converting light energy into heat energy 39.

#### Improvement of safety

One of the adverse effects of PDT is the 'always-on' phenomenon, which requires patients to be kept in the dark for a long time (usually several weeks) after treatment. An adaptive PS should be developed that can be activated in the tumor microenvironment to produce ROS to avoid low tumor specificity of PS and the 'always-on' phenomenon. Supramolecular switches were designed and synthesized based on a host guest complex containing water-soluble column aromatic hydrocarbon (WP5) and AIEgen PS (G) 40. Formation of the host-guest complex WP5, quenches fluorescence and inhibits generation of G ROS. Binding sites between G and WP5 change through shuttle motion in acidic environments due to the pH responsiveness of WP5. Therefore, fluorescence and ROS production of host-guest complexes can be turned on at pH 5.0. Tumors thrive in acidic environments; therefore, this switch with good pH responsiveness and stability can be used in cancer therapy.

#### Stimuli-responsive PS activation

When the nanoparticles loaded with Ps reach the tumor tissue, the Ps can be activated by the stimulation of tumor microenvironment. In this case, the main factors of activating Ps are: pH, H_2_O_2_, adenosine triphosphate (ATP), and temperature. [PHC] PP@HA was assembled from PDA, CE6 and hemoglobin in the ratio of 1:2:4. Compared with normal tissue, the pH value of tumor tissue was lower. When the pH value was normal, the compound aggregated and decomposed into single up transformed nanoparticles in the endometrium / lysosome of tumor cells. At the same time, polychloride e6(Ce6) decomposes into an extinguished state, and PS can be activated effectively 41. Due to rapid growth and insufficient blood supply, tumor tissue is prone to hypoxia. Based on the difference between podophyllotoxin and normal tissues, a novel podophyllotoxin prodrug (POD-PEG) can be synthesized by combining podophyllotoxin with oxalate bond of poly (ethylene glycol)(n) mono methacrylate, which can self-assemble into stable nanoparticles. Oxalate bond can react with endogenous H_2_O_2_ in tumor to produce O_2_, which can alleviate tumor hypoxia and provide more molecular oxygen for PDT when nanoparticles are degraded and activated 42. It has been confirmed that the concentration of ATP in tumor tissue (>100 μm) is higher than that in normal tissue (1-10 nm), which provides the possibility for ATP to selectively activate PSs. Gao et al. first synthesized a macrocyclic amphiphile based on guanidinium-modified calix 5 arene pentadodecyl ether (GC5A-12C), which co-assembled with 4-(dodecyl oxy) benzamido-terminated methoxy poly(ethylene glycol) (PEG-12C) and PSs to form NPs. Nanoparticles accumulate in tumor tissue through EPR effect and are replaced by ATP, which is released with the recovery of fluorescence and photoactivity 43. Hydrogel is thermo responsive self-healing. After adding polydopamine nanoparticles (PDA NPs), the SP-(DMAEMA-co-HEMA-AA)/PEI/PDA-NP nanocomposite hydrogel presented phase change and volume shrinkage under NIR irradiation. The effect of photothermal effect of PDA NPs on the temperature of hydrogel in the corresponding region is increased by near infrared laser irradiation. Because of the hydrophobicity-hydrophobicity transition of hydrogels, DOX molecules with antitumor activity are extruded from hydrogels at temperatures above their lower critical solution temperature (LCST), thus the tumor cells are inhibited by the internal stress of the shrinking hydrogels 44, 45.

## Targets of PSs

PSs for CRC can achieve the requirements of precision medicine through multiple levels of targets. At present, the targets for PSs diagnosis and treatment are mainly divided into three types: cell, organelle and molecular targets.

### Cellular level

PSs regulate autophagy of cells, specifically by acting on target cells, affecting specific targets in cells, and inducing apoptosis or necrosis of target cells 46. Cancer stem cells are the most ideal target, and PSs can play a role before or in early stages of disease effectively. Jun Ki Kim *et al*. reported that GFP-Lgr5 + stem cells located on the surface of the colon cavity are near the center of CRC. Targeted therapy for this cell type has potential preventive effect on tumor growth, especially for high-risk population of CRC 47.

### Organelle level

**Figure 1:**
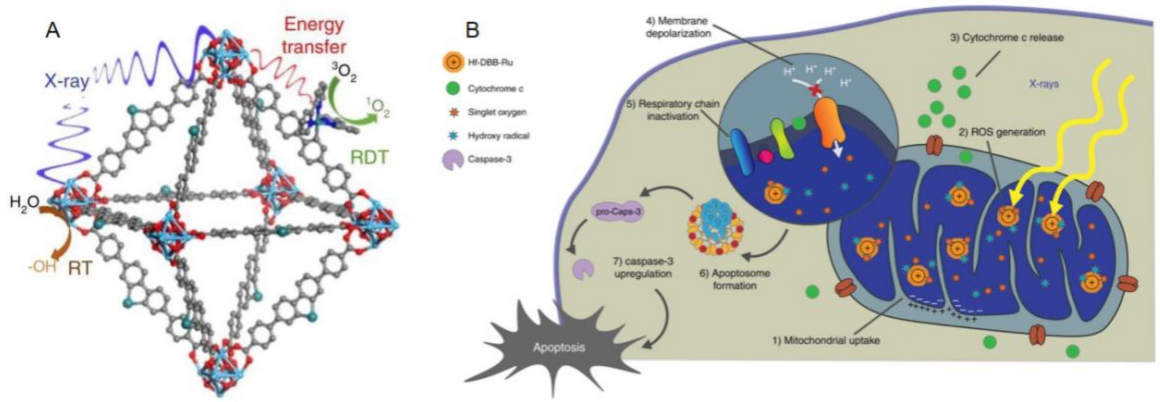
** Structure and Function mechanism of nMOF. (A)** Schematic showing the RT and RDT process enabled by Hf-DBB-Ru.6 **(B)** The process of Mitochondria-targeted RT-RDT mediated by Hf-DBB-Ru 49.

Nano drugs have the ability to kill tumor cells by initially targeting organelles, resulting in a series of subsequent reactions 48. By targeting specific receptors of a specific organelle, corresponding signaling network is activated, then the protein expression and activation level of the signaling pathway are up-regulated or down-regulated. Further, the cell death pathway can be initiated to achieve the purpose of cancer treatment by killing cancer cells. Numerous researchers have devoted themselves to the research and development of drugs targeting the organelles of CRC based on this mechanism. Kaiyuan Ni *et al*. reported a mitochondrion targeted metal organic frameworks (nMOFs) named Hf-DBB-Ru, which induces the apoptosis of tumor cells in mouse model of CRC by depolarizing the mitochondrial membrane^49^** (Figure 1)**. Sharmin akter *et al*. explored the lysosomal targeted talaporfin sodium loaded with inactivated virus particles—Laserphyrin®-HVJ-E (L-HVJ-E), which induces apoptosis and necrosis of human prostate cancer cell line PC-3, depending on light dose and concentration^50^. Modified iridium (III) protein complexes have excellent performance of aggregation in endoplasmic reticulum at low concentration 51. Most of the targeted PSs have good localization ability in organelles, but only a few can reach the nucleus. Wen Pang *et al*. reported that the effective carbon point (C point) with intrinsic nucleolar targeting ability can be used as PS to improve therapeutic performance under low dose PS and light irradiation 52.

### Molecular level

Advancements in molecular biology technology have resulted in development of application prospects for prevention, diagnosis and treatment of CRC by targeting specific molecules or molecules different from normal tissue expression. These molecules include almost all kinds of substances molecules in the whole entire process of protein expression, such as genes, RNA and proteins. Here, in this review, we discuss common molecular targets for CRC.

#### Genetic targets

TP53 Inactivation of TP53 is one of the common causes of cancer, and the inactivation of tyrosine kinase can cause the inactivation of TP53 through the Wnt signaling pathway, which will further enhance the invasion ability of CRC cells. Deterioration of CRC can be slowed down by increasing the expression efficiency of the two genes 53.

##### BRAF

The Ras / Raf / MEK / ERK signaling cascade is known as mitogen activated protein kinase (MAPK) pathway, and BRAF is a representative member of the RAF family. Abnormal activation of this pathway may lead to mutation of the BRAF gene, thus inhibiting its antitumor effect. Upregulation of the expression of BRAF gene by targeting is a potential target for the treatment of CRC 54.

#### Protein targets

##### PEG2

**Figure 2:**
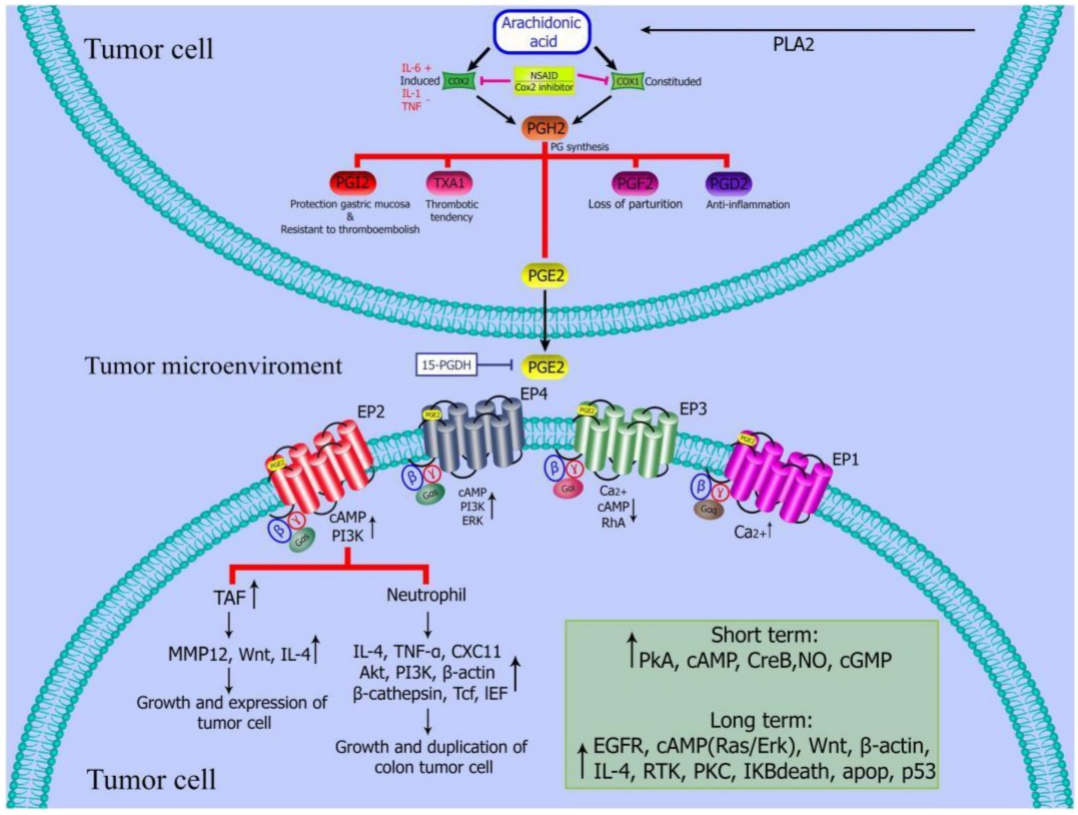
** PGE2 regulates tumor microenvironment.** PGE2 inhibits the activation of dendritic cells and B cells, and induce the activation or formation of M2 polarized macrophages, myeloid suppressor cells (MDSCs) tumor associated fibroblasts and mast cells 55.

Colon cancer can be induced by the increase in the prostaglandin level, especially prostaglandin E2 (PEG2). Targeting COX-2 / PGE2 / EP receptors may be considered as an effective treatment strategy for colon cancer. For example, NSAIDs can reduce the expression of PGE2 by administration of enzymes that inhibit Cox to reduce CRC 55 (**Figure** 2).

##### AEBP1

Overexpression of adipocyte enhancer binding protein (AEBP1), a transcriptional repressor, is correlated with the occurrence and development of tumors. Therefore, this protein can be used as a molecular marker for targeted therapy 56.

##### Bcl-2

**Figure 3:**
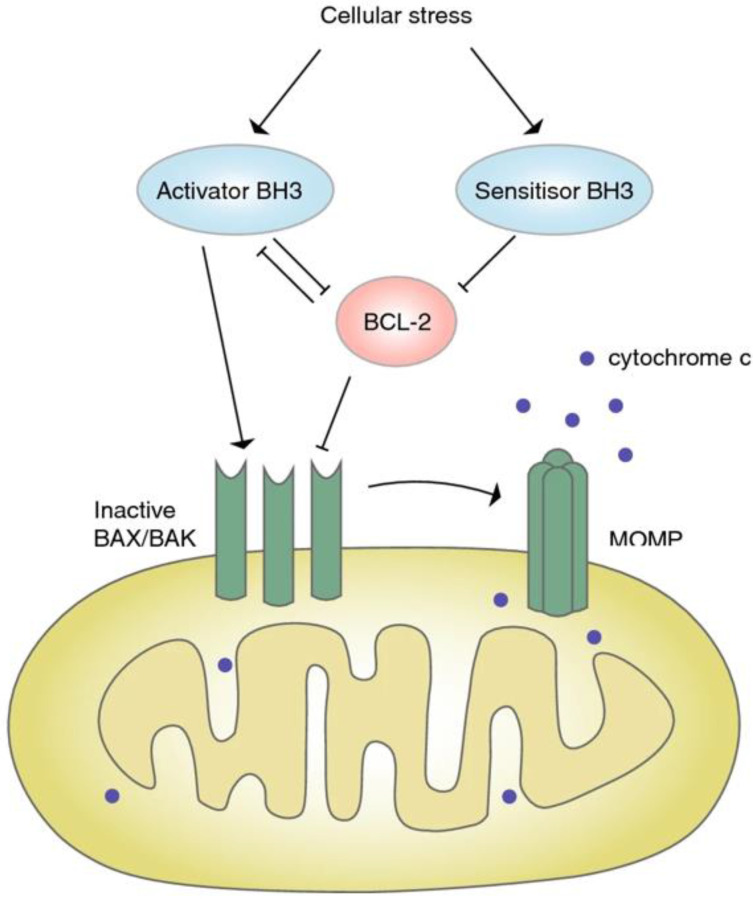
**Signaling pathway of BCL-2. Under the pressure,** the expression of BH3 increased, which further blocked the expression of Bcl-2, inactivated Bax/Bak, and resulted in the closure of MOMP and apoptosis 57

Bcl-2 is an anti-apoptotic protein that is closely associated with the occurrence of several cancers. BH3 mimics can specifically inhibit the expression of Bcl-2, and change the imbalance of apoptosis of CRC cells 57 (**Figure** 3).

##### PD-1/PD-L1

Checkpoint block is one of the important mechanisms of tumor immunotherapy. Drugs targeting PD-1/PD-L1 checkpoint shows a relatively long-term tumor growth inhibition. The combination of photosensitive material and related monoclonal antibody shows significant inhibitory effect on CRC cells 58.

##### EGFR

**Figure 4:**
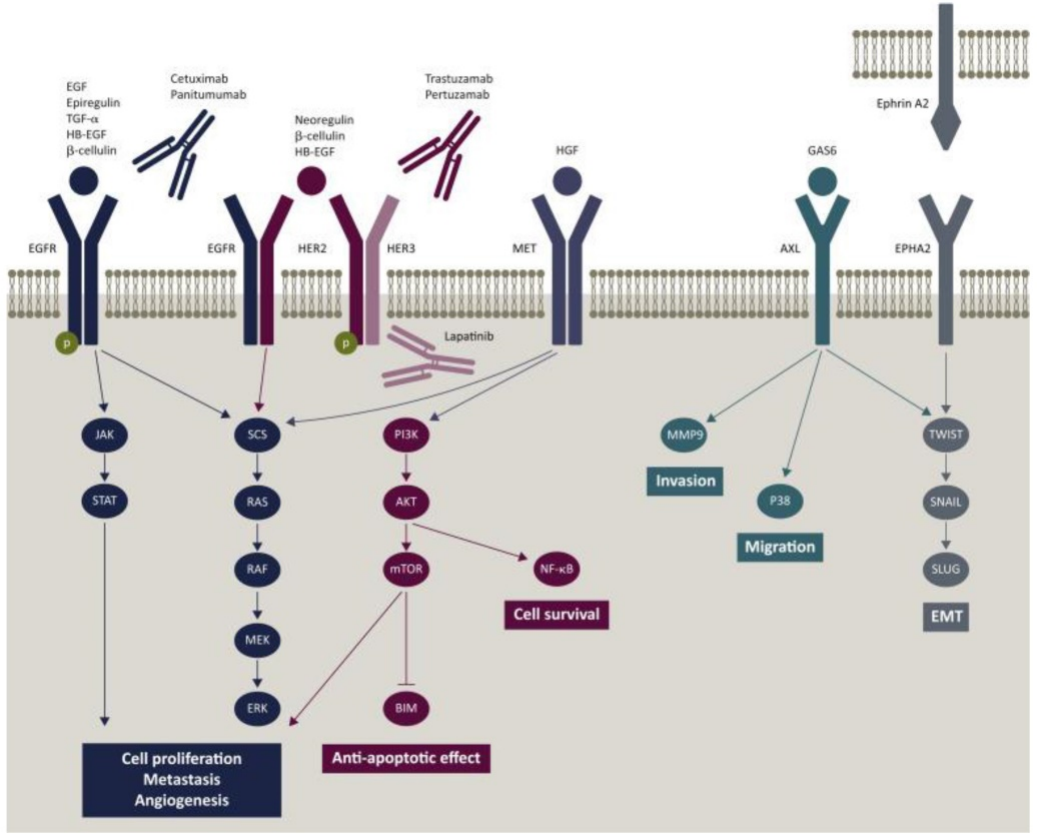
Epidermal growth factor receptor (EGFR) signaling pathway and potential mechanism of resistance to cetuximab and panitumumab 59.

Given that CRC is easy to metastasize, the strategy of inhibiting malignant transformation is more reliable. Inactivation of EGFR or blocking of the EGFR-related pathway is an effective approach for diagnosis and treatment of CRC 59 (**Figure** 4). Cetuximab and Panitumumab have been widely used for the treatment of metastatic CRC. Cetuximab is a chimeric (IgG1) monoclonal antibody (mAb). It blocks the binding of endogenous ligands by binding to the extracellular domain of EGFR, which significantly inhibits the growth of tumor cells. In addition, it may also have immune-mediated antitumor effects, such as antibody dependent cell-mediated cytotoxicity 60.

## Photodynamic therapy

### Targeted transport process and mechanism of PS

Several recent studies explored the mechanism of PDT at the cellular and molecular level. The basic process of PDT is that PS is injected into the body, and is accumulated in tumor tissues or cells through passive or active targeting and the lesion is irradiated with specific wavelengths of light. Irradiation then stimulates PSs to produce ROS, leading to tumor cell death (through apoptosis, necrosis, and excessive autophagy processes 61). Accumulation of PS in tumor cells or tissues is achieved by passive or active targeting *in vivo*. Passive targeting refers to specific accumulation of PS in tumor tissues due to the difference in physiological conditions between tumor tissues and normal tissues after entry of PS into the human body. The differences between tumor cells and normal tissue cells are as follows: 1. Metabolism of tumor tissue is more vigorous compared with that of normal tissue, and pH is lower compared with that of normal tissue; 2. Tumor tissue contains more lipoprotein; 3. The space between tumor tissue and normal tissue is larger; 4. Tumor tissues are characterized by more macrophages and poor lymphatic circulation. Active targeting is the process of combining PS with tumor cell proliferation, metabolism, vascular, and other specific factors (such as tumor antigen, serum protein and epidermal growth factor 62) to achieve PS specific recognition of tumor tissue. PS nanoparticle leles, light, and ^1^O_2_ are the three important factors of PDT. PDT achieves synergistic effect through combination with traditional therapy and has a very broad application prospect in clinical treatment of tumor.

### Photodynamic molecular reaction of PS

**Figure 5:**
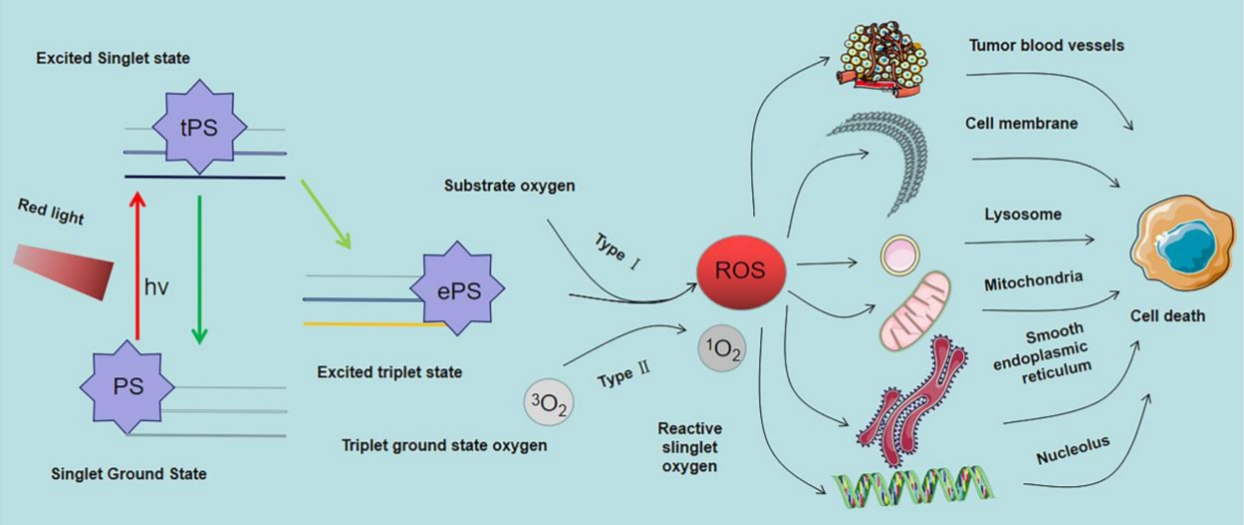
Mechanism of photodynamic reaction and targets of ROS singlet ground state. The Ps transits from singlet ground state to triplet ground state and produces ROS through type I reaction, and ROS can mediate tumor cell death by acting on various organelles and tumor microvessels.

After entry of the PS into the cell or tissue it is absorbed by light of specific wavelength. The absorbed energy changes the PS from ground state to singlet excited state, and then to triplet excited state through intercellular channeling. The triplet excited state reacts with the substrate to produce ^1^O_2_ and ROS. These products are cytotoxic; therefore, they kill tumor cells directly or indirectly. This process is mediated by the light in the PDT reaction that can be generally divided into type I and type II reactions. However most PDT reactions are type II reactions 63. As shown in Figure 5, In type I reactions, triple excited PS nanoparticles react with cell membrane or biological macromolecules to transfer a hydrogen atom or electron to form free radicals. These free radicals then interact with oxygen to generate ROS (such as superoxide anion, hydroxyl radical and hydrogen peroxide) to kill targeted cells. In type II reactions, triple laser PS directly transfers energy to oxygen molecules to generate free radicals. ^1^O_2_ (also a kind of ROS, different from type I reaction products) has high cytotoxic effects, and is a highly reactive substance that reacts with oxygen-sensitive groups in biomolecules (such as unsaturated fatty acids, proteins and nucleic acids) to cause oxidative inactivation and significant damage to tumor cells. In contrast to other ROS, the ^1^O_2_ lifetime is low, approximately 10-320 ns the diffusion and migration ability in cells and tissues is weak, approximately 10-55 nm, which limits its action range 64. These findings imply that the location of activated PS determines the area of PDT causing direct tissue damage 65.

### Damage mechanism induced by PDT

Studies report that the mechanism of action of PDT at the molecular level indicates is through PSs aggregating in tumor cells under the action of relevant light sources. After aggregation, ^1^O_2_ and ROS are produced, thereby destroying cell structure, causing oxidative damage to cells, and ultimately leading to cell death 66. At the subcellular level, PSs selectively accumulate in cell membranes, mitochondria, lysosomes, and other subcellular structures, thus directly damaging these cell structures. Damage of organelles to a certain degree results in cell death. In addition, these activities induce programmed cell death. Different types of PSs have different preferential accumulation sites, and the same PS can cause orderly or simultaneous damage to multiple organelles.

#### Mitochondrial injury

**Figure 6:**
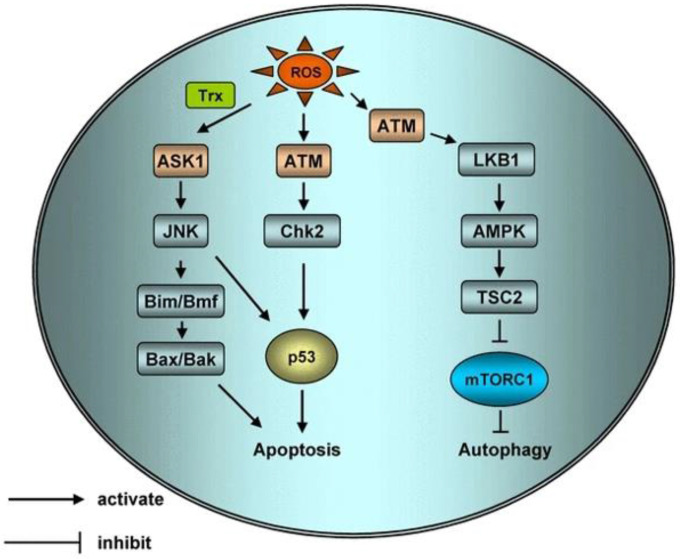
Schematic of apoptotic and autophagic signaling upon ROS. ROS can activate Apoptosis Signal Regulating Kinase 1 (ASK1) by activating sulfur peroxide protein, ASK1 can regulate protein phosphorylation of JNK, ASK1 can activate Bax and Bak or increase the expression of p53 through Bim and BMF to initiate apoptosis. H_2_O_2_ activated ATM independently by MRN/ser-1981. Activated ATM induces apoptosis and autophagy through CHK2/p53 and LKB1/AMPK/ TSC2/ mTORC1 pathways respectively 69.

Mitochondria are the most vital organelles in production of cellular energy. Mitochondria host a series of vital life activities such as cellular aerobic respiration and tricarboxylic acid cycle. Several types of PSs target mitochondria. Fluorescence microscopy shows that the second-generation PS, 5-ALA is mainly distributed in mitochondria, and its mechanism of action is by changing mitochondrial membrane potential. Some mitochondria are damaged after 5-ALA therapy, cristae of mitochondrion become invisible, and vacuoles and swelling are observed. ROS produced by PS decreases mitochondrial membrane potential and resulting in depolarization, causes disappearance of mitochondrial cristae, and expansion leading to rupture of the outer membrane. Destruction of mitochondrial structure interrupts respiratory activity and oxidative phosphorylation and inhibits calcium uptake. In addition, apoptotic effectors in the mitochondrial intermembrane space are released to induce apoptosis. For example, cytochrome C, apoptosis-inducing factor and calcium ion^67^ are released into the cytoplasm, acting on calcium-dependent proteins or cause destruction of cells by activating apoptotic signals such as caspases, Bcl_2_ and Bax, etc. Chromatin in the nucleus is destroyed, resulting in cell dysfunction, generation of apoptotic bodies, and apoptosis 68 (**Figure** 6).

#### Lysosomal damage

PSs targeting lysosomes mainly cause cell death through apoptosis. Photodynamic action triggers primary damage of lysosomes, activates acid sphingomyelinase, and releases ceramide, leading to cell apoptosis 70. The pH of lysosomes in tumor tissues is lower compared with that in normal cells, thus it stimulates pH-activated PS. Therefore, after PSs are transported into lysosomes, they are activated, causing lysosomal membrane damage ultimately inducing apoptosis 71. Furthermore, lysosomal damage can also indirectly trigger mitochondrial apoptosis by transferring some apoptotic proteins between organelles.

#### Cell membrane damage

Damage mechanism of PS targeting cell membranes is through inducing apoptosis, Initiation of apoptosis occurs through two pathways. The extracellular pathway refers to the combination of PSs with tumor necrosis factor gene family death receptors on the cell membrane and membrane surface. This combination activates transforming protein, activates caspase-8 apoptotic protein, and cascade reaction leading to apoptosis. In the internal pathway PS induces apoptosis by recognizing receptors on the surface of the cell membrane, enters cells, and binds to the mitochondria and lysosomes, etc. through the internal pathway of organelles 72.

#### Nuclear and genetic material damage

PSs, such as phenocyanines and HPD, bind to the nuclear membrane after enter into cells. PSs get to hinder cytoplasmic separation and cause significant damage to chromatin, leading to production of multinucleated cells and malformed nuclei. Chromosomes and chromatin are implicated in different stages of cell mitosis and cells in different stages have varied sensitivity to PS. S and G1 phases are more sensitive to PS, whereas the M phase is less sensitive to PS. PS attached to the nuclear membrane can directly damage the nucleus and DNA during mitotic metaphase 73. Analysis of the ultrastructure shows that guanine in DNA is deformed, leading to changes in DNA single-strand breaks, chromosome aberrations, sister chromosome exchanges and DNA-protein crosslinks, etc 74.

#### Damage to blood vessels in tumor tissues

Tumor vascular tissues have different physiological characteristics compared with normal tissues, such as abnormal endothelial morphology, discontinuity, irregular shape, enlarged lumen, increased leakage, partial loss of adventitia and basement membrane, and tumor cell embedding 75. ^1^O_2_ in the photodynamic action of PS causes acute damage to microvessels at the tumor site, resulting in vascular obstruction, which in turn causes cell necrosis due to inadequate access of tumor cells to oxygen 76.

#### Involvement of the immune system

Immune system PDT induces local tissue edema, which is a manifestation of immune system involvement in regulation. Korbelik *et al*. 77 treated normal BALB/c mice and immune deficient mice with PDT and reported that recurrence rate of tumors is higher in immune deficient mice. This finding indicates that the body requires an immune response to clear remaining cancer cells after treatment. PDT promotes release of immune inflammatory factors, stimulates and induces immune response. Gollnick *et al.* 78 report that tumor cells after PDT treatment have immunogenicity and can stimulate the specific immune response. Combination of PDT and immunotherapy-photodynamic immunotherapy high potential for treatment and control of malignant tumors as well as in prevention of tumor metastasis and recurrence.

### Application of classical PS in treatment of CRC

**Figure 7:**
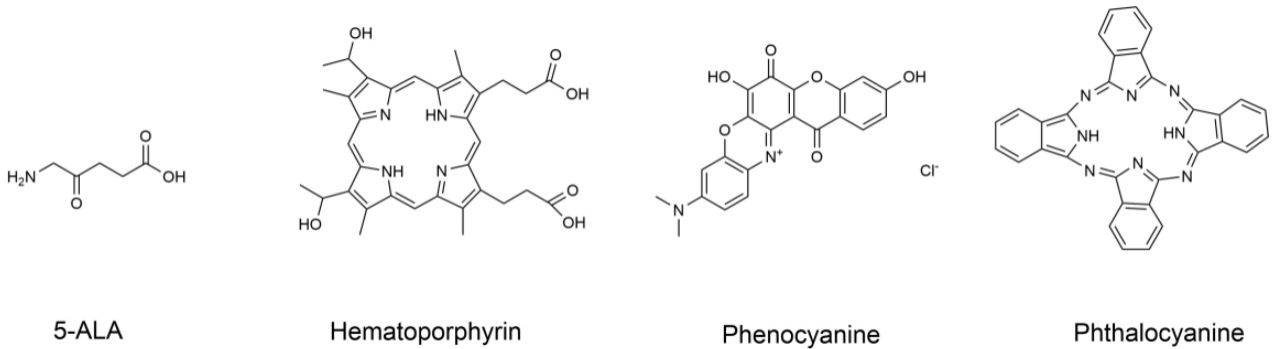
Molecular structure formula of some PSs.

Since the discovery of the photodynamic effect of some dyes and crude hematoxylin in 1920, advances in development of PSs have been achieved resulting in three generations of PSs. PSs are modified by combination with tumor cell targeting specific groups and carriers, such as monoclonal antibodies, polypeptides, somatostatin, folic acid and LDL, etc. In 1993, hematoporphyrin as shown in Figure 7 was first used to treat bladder cancer in Canada and was further used for treatment of the esophagus, lung, stomach, and cervix tumors 79.

HPDs have high efficacy; however, studies report that HPDs have severe side effects. HPDs accumulate in the skin after entering the body of patients, and the clearance of drugs from the skin is slow. Therefore, patients need to be protected from light for 4-6 weeks after using drugs. These side effects greatly limit the clinical application of HPDs.

5-ALA is a PS precursor with high activity, high fluorescence intensity, convenient administration, safety, and skin allergic reaction, which can generally be eliminated within 48 h 80. A study by Luo Li ping,* et al*. 120 CRC grouped patients into two groups to undergo conventional chemotherapy. The chemotherapy regimen was PF regimen [fluorouracil and cisplatin]^66^, where patients were subjected to 12 cycles of chemotherapy, and underwent PDT treatment one week after chemotherapy. Patients in the control group were treated with HPD injection as the PS approach, whereas the PS in the observation group was 5-ALA injection. The two groups were treated separately. The experimental results showed that the survival time and rate of the observation group were significantly higher than compared with those of the control group, and 5-ALA had a significant role in treatment of CRC.

## Photothermal therapy

### Mechanism of PTT

Photothermal therapy is a treatment method that uses materials with high photothermal conversion efficiency to inject them into the human body. The approach used targeted recognition technology to gather near the tumor tissue, and converts the light energy into heat energy under irradiation of NIR light to kill the tumor. Tumor cells have lower heat tolerance compared with normal tissue cells, therefore the balance has less impact on normal cells 81.

### PTT injury mechanism

#### Direct cell damage

After the ablative agent enters the tumor cell, it generates heat under irradiation of NIR increasing the temperature of the tumor cell. High temperatures change permeability of the cell membrane, affect the sodium-potassium pump and protein ion channel, and affect selective permeability of the cell. In addition, this phenomenon causes destruction of cell membrane structure, disorganizes the cytoskeleton, impairs cell function, and leads to cell death. Furthermore, high temperatures can change structure of chromosomes and inhibit their synthesis, thus affecting DNA repair and causing damage to genetic material 82.

#### Damage to blood vessels in tumor tissue

Tumor tissues and normal cell tissues have significant differences in physiological morphology. Tumor tissues are characterized by an abnormal growth of blood vessels, structural disorders, sinusoid formation when capillaries are compressed low heat response and large blood flow. Generation of heat by the ablative agent results in low blood flow inside the tumor tissue, increases resistance, lowers heat dissipation levels, and significantly increases temperature, resulting in insufficient cell oxygen and pH value lower than normal, ultimately inducing cell apoptosis^83^, blood vessels of normal tissues expand under high temperature, blood flow is accelerated, blood circulation is good, and heat dissipation is rapid. PTT affects repair of tumor vascular by inhibiting tumor-derived vascular endothelial growth factor and its expression. These changes hinder endothelial cell proliferation and inhibit tumor growth and metastasis.

### Types of ablation agent

Ablative agents for PTT use should have good biocompatibility, good photothermal conversion efficiency, and good biological targeting. Nanoparticles have selective photothermal conversion efficiency and can passively accumulate in tumor cells 84. Nano drugs have several advantages in targeting tumors in tumor treatment, therefore, nanoparticles are widely used as ablators in PTT. Nanomaterial ablators for PTT can be divided into precious metal nanoparticles, semiconductor nanoparticles, carbon-based nanoparticles, and organic nanoparticles based on the material used for their development 14.

#### Noble metal nanoparticles

Surface plasmon resonance (SPR) of noble metal nanomaterials is the basis for their photothermal conversion ability. Absorption wavelength and photothermal conversion efficiency of metal nanoparticles depend on the SPR effect and are adjusted according to the size and structure of these nanoparticles 85. In 2017, Hosseinzadeh *et al.* used gold nanosphere particles, which had a maximum absorption wavelength of 630 nm and was in the visible wavelength range to target MUC1 aptamer *in vitro*. Gold nanoparticles can reduce the active migration ability of CRC cells through photothermal effect, however, the absorption peak of visible radiation limits the application of pure gold spherical nanoparticles 86. The absorption peak of gold nanoparticles can be adjusted by hybridizing them with other materials. Gold nanoparticles have the best PTT efficacy in the NIR region 87. The absorption peak of gold nanorods can be adjusted to the NIR by adjusting the aspect ratio 21. Gold nanoshells can be used for adjustment of absorption peaks to the NIR by adjusting size and thickness 88. Gold nanoparticles have significant photothermal effects on tumor cells and tissues. However, low photostability of gold nanoparticles results in structural deformation 89, which limits application and development of gold nanoparticles. Future studies should design a gold nanomaterial structure with good photostability and high photothermal effect.

#### Carbonyl nanoparticles

Carbonyl nanoparticles have electrochemical and non-covalent bonding properties, good physical properties, significant absorption in the infrared region and are widely used as ablators. Development of carbonyl nanoparticles, such as graphene and carbon nanotubes 90 for use in PTT is currently underway. Graphene structure consists of flat carbon atom sheets whereas carbon nanotubes are three-dimensional structures made of graphene. The two materials can be combined with magnetic materials and chemotherapeutic drugs to act as ablators 91. Graham EG* et al.* combined MWCNTs with folic acid for treatment of colon cancer cells. Combination of carbonyl nanoparticles with folic acid, increased the affinity of the structure to CRC cells increased by four-fold to five-fold, whereas activity against CRC cells decreased by 50%. Markovic* et al*. 92 analyzed the type of 20 cell death caused by graphene-induced PTT. Cells were found to die by necrosis and apoptosis according to their characteristics. However, studies on carbonyl nanoparticles show low photothermal conversion efficiency and require higher light intensity or wavelength. Excessive intensity and wavelength of light damages normal cellular tissues, results in easy deposition into organs such as the liver, kidney, and skin, causing granuloma formation and leading to the development of cysts and organ damage 92.

#### Semiconductor nanoparticles

Semiconductor nanoparticles have the advantages of low cost and cytotoxicity unlike noble metal nanoparticles. Copper chalcogen nanoparticles are the main types of semiconductor nanoparticles. Photothermal conversion efficiency of copper-based nanoparticles can be improved by adjusting the size and shape. In Hu 93 and other experiments report synthesis of Cu-based nanoparticles with uniform 3D floral morphology. In these studies, a hydrophilic copper substrate-like nanomaterial was developed. Its photothermal conversion efficiency was up to 25.7%, which is slightly higher compared with that of gold nanomaterial under the same wavelength of irradiation. Studies report use of semiconductor nanoparticles have been used for treatment of CRCs with high effectiveness. Copper sulfate nanocomposites (CuSO_4_ NCs) reduces viability of CRC cells after NIR irradiation 94. Polyethene glycol-coated copper nanowires (PEGylated CuNWs) induce tumor cell necrosis and inhibit tumor growth in CRC subcutaneous tumor-bearing mice by intratumoral injection and NIR irradiation 95. In a study by Hessl *et al*^96^, the synthesized copper selenide nanocrystals induced tumor cell death under NIR irradiation. Shortcomings of semiconductor nanoparticles are similar to those of noble metal nanoparticles. They have low photothermal conversion efficiency and require a high intensity of light irradiation. These particles can easily cause damages to normal tissue cells.

#### Organic nanoparticles

Organic nanoparticles have better biocompatibility and biodegradability compared with inorganic nanoparticles 97. Therefore, high numbers of organic nanoparticles are used in PTT. Organic nanoparticles under evaluation include NIR dye-based nanomicelles, porphyrin liposome nanoparticles 98, small organic nanoparticles and organic polymer nanoparticles. NIR dye-indocyanine green nanoparticles can be excreted through urine, thus reducing cytotoxicity caused by drug accumulation. In addition, nanoparticles carrying liposomes and proteins can be degraded *in vivo*. Furthermore, organic nanoparticles can be combined with functional molecules to prepare better nanoparticles and can effectively slow growth rate of tumor volume, causing tumor tissue necrosis. Sensitivity of photobleaching is a defect of organic photothermal agents. Absorption capacity of organic photothermal agents decreases rapidly and cannot accept long-time light source irradiation under prolonged irradiation.

## Application of imaging examination and PDT / PTT in tumors

Due to the diagnosis of CRC and detection imaging methods, there is a big gap result in a lot of problems in the early diagnosis of CRC. Phototherapy, a promising method for targeted treatment of tumors, is effective in treatment of CRC. Combination of PDT and PTT with various imaging examinations of CRC ensures early treatment of patients thus reducing cases for surgery requirement 99. Image-guided therapy can integrate various information to infer distribution and metabolism of PSs 100. In addition, integration of imaging and treatment into single-imaging guided multifunctional cancer treatment platform improves treatment efficiency and safety of this approach 101.

“Therapeutics” refers to combination of therapeutic model and diagnostic imaging in one system, among which noninvasive phototherapy has attracted extensive attention in recent years.

### Combination of fluorescence imaging with PDT / PTT for tumor treatment

In the process of high-energy fluorescence, a kind of fluorescence signal with different orbital wavelengths is produced when high-energy electrons are irradiated to ground state. Fluorescence imaging is collection and recording of the intensity of emitted light signal in a certain range. It is characterized by high spatial-temporal resolution and short imaging acquisition time. Advantages of fluorescence imaging include high stability, low toxicity and low cost. PS used in PDT and PTT have strong absorption in the NIR, which can convert the absorbed NIR light energy into fluorescence, ROS, and heat. Fluorescence imaging has the characteristics of tissue spontaneous fluorescence and light scattering in the “NIR optical window” 102. A variety of multi-functional diagnosis and treatment systems for cancer NIR fluorescence imaging and combined phototherapy are currently available. Naphthalocyanine (NC) has a strong absorption near 800 nm, which provides a broad application prospect for fluorescence imaging and combined phototherapy of deep tumors^103^. However, due to the planar structure of NC molecules, their solubility in water and aggregation are limited. Sigh *et al.* integrated SiNC and IR780 dyes into mesoporous silica nanoparticles and explored their photothermal ability under light irradiation and their potential for use as nanoparticles for fluorescence imaging and PTT 104.

Effective packaging strategy reduces aggregation of SiNC molecules, retains the fluorescence signal of NIR, stabilizes the characteristics of PDT and PTT, and enhances the water solubility 103. Application of SiNC-NP in development of NIR can be evaluated by recording the strong fluorescence signal in the tumor area. Previous studies report advances in development of fluorescence imaging for PTT and PDT guidance. The methods have a spontaneous fluorescence background and limited penetration depth compared with other imaging technologies. Moreover, NIR fluorescence NPs has low quantum yield and limited fluorescence signal 105, 106.

### Combination of photoacoustic imaging with PDT / PTT for tumor treatment

Photoacoustic imaging (PAI) is a new non-invasive and non-ionizing biomedical imaging method. Phototherapy, combined with diagnostic photo imaging, has great potential for precision tumor therapy. PAI provides tissue imaging information at a certain depth of penetration and has high image contrast and spatial resolution 107. PAI is often combined with PTT because photoacoustic signals originate from photothermal conversion generated by the absorption and excitation of PS 20. PAI is a combination of photoacoustic microscopy (PAM) and photoacoustic tomography (PAT)^61^. PAM and photoacoustic endoscopy can be used for diagnosis of CRC. However, due to the low imaging speed, PAT has a larger penetrating field and deeper area compared with PAM 105. Shi *et al*. prepared NIR light-harvesting fullerene-based nanoparticles (DAF NPs) with good biocompatibility for photoacoustic imaging-guided tumor PDT and PTT by nano precipitation method 106. In addition, Pu *et al*. used PEG-b-PPG-b-PEG to encapsulate fullerene in semiconductor polymer nanoparticles to prepare a molecular diagnostic system for PAI guidance 108. The findings of these studies showed that photoacoustic and photothermal properties of SP nanoparticles were enhanced after encapsulation with fullerene.

### Combination of nuclear magnetic resonance imaging with PDT / PTT for tumor treatment

Nuclear magnetic resonance imaging (NMRI) is used to detect emitted electromagnetic wave in external gradient magnetic field, obtain the position and type of atomic nucleus in study material, and draw the internal structure image of the object using the energy released from a specific substance to attenuate in different materials. Advantages of MRI including having no harm to the human body and it is a safe, fast and accurate clinical diagnosis method. It provides rich diagnostic information, such as ability to distinguish benign and malignant tumors. Phototherapy induces apoptosis or necrosis of tumor tissues and has low toxicity on normal tissues. MRI is used to detect confirm the location and size of the tumor *in vivo* and provides diagnostic assistance and real-time monitoring for phototherapy. Several multifunctional composite nanoparticles with photothermal effect and MRI have been developed, which can be used in diagnosis and treatment of tumors.

As a common compound, porphyrin is a pioneer in photodynamic cancer treatment and MRI 109. The application of porphyrins and their metal derivatives in molecular imaging is a hot topic in recent years. Porphyrin, a first-generation of PS serves as a carrier and has high drug release ability in target cells, therefore it is effectively used in PDT of tumor. Tetranuclear gadolinium (III) porphyrin complexes reported by Luo *et al*. 110 can be used as therapeutic drugs for MRI and PDT.

**Figure 8:**
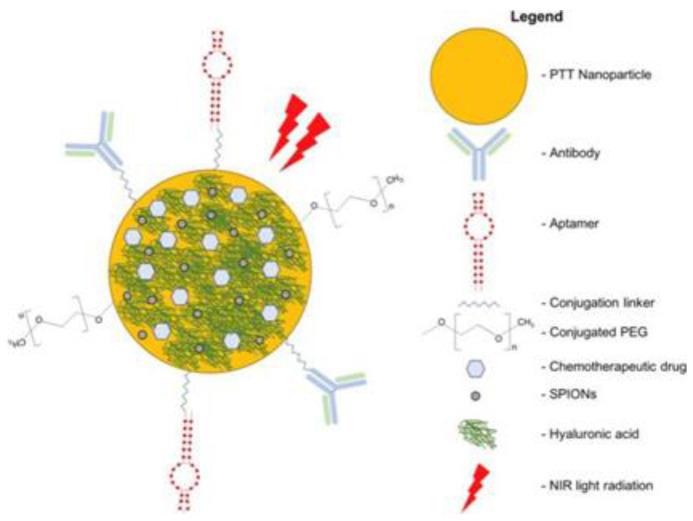
Schematic diagram of multimodal targeted PTT nanomedicine.

In recent years, research on MRI contrast agents has developed rapidly. NMRI contrast agents can be divided into two categories: paramagnetic and superparamagnetic materials 111. In this review, nano drugs, refer to using nanometer carrier or nanoparticles modified photosensitive material. They make use of superparamagnetic iron oxide nanoparticles (SPIONs) property to improve magnetic resonance imaging and multimode PTT nano drugs in addition to field agent. Furthermore, the agent can provide tumor imaging and are used for combination with chemotherapy drugs 112 (Figure 8). Although now most studies are still in preclinical stages, further clinical environment research on PTT nano drug treatment of CRC should be carried out. Requirements for contrast media are also strict, and include being safe, having no harm the body, and can be improved to reduce toxicity. Combination of specific coordination metal ions with porphyrin, then the ability of RF pulse can be affected, which can be used as MRI contrast agent, the basic standard of MRI contrast agent is to affect RF pulse. Copper, iron, manganese and other metals are inserted into porphyrin respectively. Studies report that manganese complex is the best contrast agent. Therefore, manganese-based contrast agents are prepared by combining manganese with tetraaryl porphyrin cavity 15. Although some contrast agents have been approved for clinical use, no contrast agents are reported for treatment of malignant tumors.

Multimodal nanomedicines combine different therapies such as NIR light-absorbing nanoparticles to mediate PTT and conventional chemotherapeutic drugs (ie, Doxorubicin, SN38, and Oxaliplatin). Nanomedicines can be functionalized with SPIONs to Facilitate Magnetic Resonance Imaging and Polyethylene Glycol (PEG) to improve biocompatibility and prolong systemic circulation. Cancer-targeting ligands such as antibodies, aptamers, and Hyaluronic Acid (HA) can be conjugated onto nanomedicines to improve delivery and uptake of nanomedicines in cancers 112.

### Combination of nuclear imaging with PDT / PTT for tumor treatment

Nuclear imaging technology is a new technology that combines nuclear technology science with modern image theory, including positron emission tomography (PET), single-photon emission tomography (SPECT), X-ray tomography (XCT), nuclear magnetic resonance computed tomography (NMR-CT), and Compton scattering tomography (CST). PET and SPECT with nuclear imaging function are used for tissue imaging and have a deeper tissue penetration effect compared with fluorescence imaging. Imaging information is provided to PET and SPECT by detecting radioactive signals of radioisotopes 4. SPECT uses a γ camera to rotate around the human body to sample the whole body or a limited part of the body to achieve tomography imaging. The principle of PET is based on the positron and electron annihilation effect to detect photons formed after annihilation. Cancer cells can be detected with special capabilities of PET, such as radioisotope labeling on PET, and pharmacokinetics of PTA can be monitored by PET or SPECT imaging technology.

Dynamic PET imaging for real-time PDT monitoring is a promising method for study of tumor response. It can detect uptake of PS in tumor sites throughout the treatment period. Real-time dynamic PET is used to distinguish two main mechanisms of PDT: direct cell death and tumor angiogenesis 113. Dris Kharroubi Lakouas D* et al*. demonstrated that the absorption of 64Cu-DOTA-biotin-sav after PDT is displayed by PET imaging. SPECT imaging shows absorption of 99mTC-Annexin-V after PDT. Berard *et al.* used PET imaging based on 2-deoxy-2-[(18)F]fluoro-D-glucose(18-FDG) radioactivity to study the effect of dynamic real-time treatment 114, 115. In addition, different sulfonated phthalocyanine analogs have been used as PSs to evaluate the effect of PDT on tumor 18-FDG uptake. Studies report that direct induction of cell death by 18F-PDG was characterized by a rapid reduction of 18F-PDG uptake, followed by a return to more than 80% of the initial rate at the end of light exposure. Nuclear medicine is an essential technology in the current tumor phototherapy. Nuclear imaging not only monitors the tumor but also evaluates the uptake rate of PSs in deep tumors to improve treatment.

Although many studies have shown that imaging and PDT / PTT are widely used in tumors, many shortcomings persist, such as single-mode imaging, separate application of PDT or PTT. The outcome is often unsatisfactory due to the defects of each imaging technology and single therapy. Multimodality imaging is the current research direction to integrate the advantages of each imaging mode into one. Zhang* et al.* 100 studied the PTT-PDT dual therapy system guided by fluorescence Mr dual-mode imaging, and constructed a new core-shell system, Fe_3_O_4_ @ C @ PMOF Nanocomposites. The results of tumor-bearing mice showed that it had high tumor accumulation and low cytotoxicity.

## Synergistic therapy of PTT and PDT

Although PDT and PTT show great potential in non-invasive and selective treatment of tumors, they have shortcomings when used alone. Synergistic therapy includes combination of chemotherapy and PTT, PDT and chemotherapy synergy, PDT and PTT synergy, and PDT, PTT, and chemotherapy synergy. PDT and PTT combined therapy has a wide potential for application prospect in tumor treatment. Synergy therapy is less toxic and more effective compared with monotherapy. Therefore, several studies are currently exploring synergy therapy, according to the latest literature summary, we get **Table 1**, which compares and analyzes the characteristics of photodynamic therapy, surgical therapy and chemotherapy and radiotherapy.

### Limitations of PTT and PDT

Most PSs and ablation agents are nanomaterials. However, nanomaterials have certain limitations in phototherapy for tumors including: 1) Most PSs have a hydrophobic structure and have a small solvent degree, making it difficult to generate stable solutions. 2) These compounds are easy to stay and aggregate in the body and have certain physiological toxicity to normal tissues, therefore, targeting needs to be further improved. 3) The infrared absorption efficiency of PSs and generation effect of active oxygen under the action of NIR 116. 4) Photothermal conversion efficiency of ablative agents should be further improved. 5) Ablative agents have low light stability.

### Theoretical basis of combination therapy

Studies on PDT and PTT report that most of the newly synthesized PSs and ablation agents are nanomaterials, PSs and ablation agents need to function under irradiation of NIR, and mechanisms of cell death induced by the two are similar. Therefore, several studies are currently focusing on the combination therapy of PDT and PTT. Uses of the ablative nanoparticles are used as carrier for PSs alleviates shortcoming of water solubility and light wavelength. Furthermore, PSs can also improve the light-to-heat conversion efficiency of the ablative agent, resulting in an efficient radiation treatment system. Combination of PDT and PTT triggers photothermal effect to enhance the efficiency of PDT, thus producing a synergistic effect of photothermal effect, accelerate blood flow in the tumor, and results in delivery of more oxygen to the tumor tissue, thereby improving efficacy of PDT.

### Application of PDT and PTT Combination Therapy

PSs and ablators are combined in a variety of ways and exhibit good anticancer activity by synergizing photothermal and photodynamic pathways under the action of NIR. Seo SH *et al* 117. report that combination of PSs nanoparticles and ablator nanoparticles show improved effects. In the study, gold nanorods (GNR) and methylene blue (MB) were used as photothermal materials. Methyl blue is a water-soluble phenothiazine PS with high ^1^O_2_ yield. GNRs are used as ablators. MB-GNR prolongs NIR irradiation time, produce varieties of ROS compared with a single therapy, exhibit dual effects of PTT and PDT, and have good anticancer activity. Further, in a different study pyrrolo-pyrrole-triphenyl lamine organic nanoparticles (DPP-TPA) were synthesized for PAI and used in combination with PTT and PDT. In animal experiments, DPP-TPA was injected into subcutaneous tumor-bearing mice. Administration of synergistic therapy, the tumor disappeared completely after treatment.

In a study of Anthony J Trinidad *et al* 118, gold nanoshells were combined with macrophages to improve delivery for PTT. Macrophages containing gold nanoshells were used to separate human head and neck squamous cell carcinomas. The combination was developed by designing PTT then PS for PDT was added, then the mixture was subjected to 670 nm and 810 nm light to explore the synergistic effect of PDT and PTT. Findings of the study showed that the combined treatment reduced the viability of head and neck squamous cells to less than 40%, and PTT and PDT showed significant synergistic effects.

## Clinical application of PS in CRC

Given its low biological toxicity and excellent metabolic dynamics, PS treatment of CRC has a high safety factor, and its more favorable effect is not only can act on the lesion site of local intestinal tissue but also can induce the whole body immune response. Increasing number of studies have been conducted on accelerating the clinical application of PSs. Up to now, many PSs have been used in CRC research. Although still in the experimental stage, its powerful role has attracted wide attention.

In this review, we summarize the clinical research of PSs. (**Table 2**)

### Organic PSs

#### DC 473

A novel PS DC 473, especially the CRC cell line SW 480, was designed and synthesized by Julia Gala de Pablo *et al.* It is associated with a resonant Tunku Abdul Rahman signal from the Cytochrome C, suggesting that it may be located in or near this cell compartment 9.

#### HpD

PDT induced by Haematoporphyrin derivative (HpD) showed significant biological effects on the traditional P-glycoprotein after being regulated by verapamil, thereby effectively inhibiting the growth of CRC cell line HRT 18 *in vitro* 7.

#### Hypericin

Laura Mühleisen *et al.* studied the effect of hypericin on CRC cell line HT-29, and found that hypericin mainly accumulated in the cytoplasm, which may be in the endoplasmic reticulum, Golgi body, lysosome, and the process is dose-dependent and time-dependent. Under the condition of low hypericin concentration and short illumination time, cell antioxidant system can cope with oxidative stress. However, with increasing hypericin amount and irradiation time, the antioxidant system of cells was overloaded by ROS, and the content of oxidized glutathione was no longer sufficient. Hence, hypericin can regulate the transition from apoptosis to necrosis 119.

#### PCI of 3-17I-saporin

Considering the high expression of epithelial cell adhesion molecule (EpCAM) in human CRC cell line, WiDr, Kaja Lund *et al*. designed a compound PCI of 3-17I-saporin based on photo immuno therapy. A series of experimental results showed that the cytotoxicity induced by photo immuno therapy was stronger than that induced by phototherapy alone, which provided an idea for the combined diagnosis and treatment of CRC 120.

#### Foslip ldpdt

By detecting the potential value of foslip ldpdt in establishing a good CRC mouse model, AUR é lie Reinhard* et al.* found that it can significantly reduce the incidence of cancer associated with colitis by limiting the extravasation of monocytes and neutrophils 121.

#### Talaporfin sodium

Although the drug—Talaporfin sodium has been approved for marketing in Japan to treat early-stage intrabronchial cancer, the study by Wang* et al.* is still in the stage of clinical trials. The results of phase I and phase II clinical trials show that the drug is suitable for tumors that are not sensitive to other therapies. In addition, the team's research on the effect of the drug on hepatocellular carcinoma and metastatic CRC is also in phase III clinical trials 122.

#### Sinoporphyrin sodium

Bing Zhu *et al.* observed that sinoporphyrin sodium enhanced the concentration of HCT116 cells and nuclear fragmentation, increased the levels of caspase-3 and Bax cleavage, inhibited autophagy that promoted cell apoptosis and decreased the size of tumors 123.

#### Axial disubstituted silicon phthalocyanine

Ceren SAR synthesized the effect of axial disubstituted silicon phthalocyanine on the PDT of CRC cell line HCT116. Compared with the effect on cancer cells under the condition of no light, the toxic effect on cancer cells caused by different concentrations of photosensitive molecules was examined. It was proved that the drug could induce the corresponding cancer cell toxicity only under the condition of laser irradiation 124.

#### F_2_BOH

Andre F.S. Luz *et al*. studied the photodynamic effect of water-soluble Bacillin F_2_BOH on CT-26 mice colon cancer cells and found that the number of apoptotic bodies increased after short-term and low-dose irradiation. After long-term and high-dose irradiation, the scope of cell necrosis was larger. At the same time, the compound can be transported in a non-toxic carrier, thereby reducing the corresponding side effects on organisms 125.

#### Lu dota peptide 2

Hossein behnammanesh *et al.* studied the effect of a new ^177^Lu labeled somatostatin receptor antagonist, ^177^Lu dota peptide 2 on human CRC cell line HT-27. As a Somatostatin (SST) radio antagonist, it has high stability *in vitro* and good affinity to SSTR. Therefore, the drug has broad therapeutic prospects drug for human colorectal adenocarcinoma 126.

### Nanomaterial PS

#### NSP-HA-CD

Hu Xianli *et al.* designed a prodrug nano carrier, which integrates hyaluronic acid (HA) targeting CD44 with NLG919 HA and reductive unstable heterodimer into the supramolecular nanocomposite. Under the irradiation of NIR laser, ROS are released, and the antitumor properties of cytotoxic T-lymphocytes are further enhanced. The antitumor effect of cytotoxic T-lymphocytes is obvious in CRC cell line CT-26 127.

#### HA - PDA - Ce6

Xiaoling Wang *et al*. developed a bimodal HA - PDA - Ce6 targeting CRC as a local delivery system and PTT attribute of PDT, which has high photothermal and photodynamic dual activities and can accurately reach the tumor site through CD44 mediated endocytosis to achieve the goal of drug accumulation and penetration into deep tissues. HA - PDA - Ce6 showed the strongest antitumor effect in HCT-116 mice 128.

#### CCPs / HPPH / DOX

Weijing Yang* et al.* reported a dendritic cell vaccine that essentially uses multifunctional polymer vesicles as adjuvants in combination with chemotherapy and PDT. Both DPX and PDT in CCPs / HPPH / DOX molecules can induce ICD. At the same time, the existence of adjuvant can not only enhance the immune recognition effect of the body but also reduce the drug dosage and injection times 129.

#### Ce6tetraHA

Shin Jung *et al.* synthesized a kind of Ce6tetraHA nanocomposite and detected the fluorescence intensity of the drug on mouse CRC cell line HCT116. The drug could be delivered to the tumor site in the way of redox sensitivity and CD44 sensitivity, making the fluorescence intensity of tumor site significantly higher than that of other organs and tissues 130.

### Inorganic PS

#### 8-MOP

Magdalena bartnik *et al*. confirmed that 8-MOP could activate endogenous or exogenous apoptosis pathway by inhibiting Akt phosphorylation, thereby significantly suppressing the growth of metastatic colon cancer cell line SW620 131.

## Conclusion

With the rapid development of bioinformatics technology, the properties and functions of nanoparticles are becoming more and more extensive. The design of nanoparticle as an intelligent tool to carry PSs is one of the research hotspots of precision medicine in the 21st century. Through this operation, researchers have designed a series of biodegradable materials with good biocompatibility and targeting, such as liposomes, organic microspheres and iron oxide. These nanoparticles have achieved remarkable results in a variety of tumor diagnosis and treatment experiments. However, in clinical trials, there are still huge obstacles and challenges in the application of PDT and PTT in deep tissue tumors, for example, there is no light that can reach deep tissue, there is not enough photosensitive material accumulated in deep tissue, or the heat and ROS generated are not enough to achieve the purpose of inhibiting tumor growth. Nanoparticles carrying PS therapy is expected to become a potential treatment for cancer, including colorectal cancer. Many new technologies and strategies have been applied in this field. The surface modified nanoparticles can be well targeted to specific tissues, cells and even organelles. With the help of medical imaging, we can visualize the transport of nanoparticles in the body, and provide a process for PS to play its role. The combination of nanoparticles carrying photosensitive materials with immunotherapy and chemotherapy also contributes to the development of PDT and PTT. In the future, the research of nanoparticles will focus on the safety and effectiveness evaluation of the combination of nanoparticles and PSs. It is also one of the great challenges to combine PDT and PTT with nano medicine to achieve great biological effects by using photosensitive materials loaded with small doses of nanoparticles.

The extensive and multifaceted researchs of PSs show their great application prospect. On the one hand, due to the continuous improvement of PSs own performance and the development of its compatibility with organisms, such as more accurate biological targets and better biological effects, it will be more conducive to the diagnosis and treatment of tumor, and also cater to the medical trend of integration of diagnosis and treatment. On the other hand, with the deepening of interdisciplinary, the relationship between PS and imaging, immunology, nanomaterials and other related disciplines can be further strengthened, and the association between PS and other disciplines such as genetics and other disciplines will also be established and developed in the process of communication.

As mentioned earlier, in order to further optimize the performance of PS, it is feasible to construct nanoparticles with a variety of conjugated polymers. At present, the problem of whether nanoparticles will cause potential damage to the body has not been solved. The next generation of PS research needs to improve the biological characteristics of PS and ensure its biological safety 132. In addition, we should pay attention to all aspects of the conditions needed for the interaction between PS and organism, such as optimizing the input dose of PS, drug level in tumor, light source and tissue oxygen conditions 133. What is more needed is to change to clinical related research field or even clinical under the condition of ensuring safety 134. Perhaps, in the future, researchers can establish a large database about photothermal based nanoparticles to realize the rapid change of PS industry.

## Figures and Tables

**Table 1:** Comparison of phototherapy and other cancer treatments.

Tumor treatment	Cytotoxicity	Drug metabolic rate	Selectivity	Security	Traumatic	Synergy	Risk
Photodynamic therapy	Low	High	High	High	Low	High	Low
Surgical treatment	Secondary	Secondary	Secondary	Low	High	Secondary	High
Chemoradiotherapy	High	Low	Low	Low	High	Secondary	High

**Table 2:** Application of PS in PTT / PDT.

Number	PS	Product Category	Highest Phase
1	Padeliporfin potassium	PDT	Launched-2018
2	Aminolevulinic acid hexyl ester	PDT	Launched-2005
3	Mono-L-aspartyl chlorin e6	PDT	Launched-2004
4	Temoporfin	PDT	Launched-2002
5	Methyl aminolevulinate	PDT	Launched-2001
6	5-Aminolevulinic acid hydrochloride	PDT	Launched-2000
7	Cetuximab-IRDye-700DX	PDT Phthalocyanines	Pre-Registered
8	Tin-mesoporphyrin	PDT	Pre-Registered
9	SNA-001 (Code came)	PTT	Phase III
10	ACP-SL017 (Code came)	PDT	Phase III
11	Rose Bengal disodium	PDT	Phase III
12	Hypericin	PDT	Phase III
13	inCVAX	PTT polymers	Phase II/III
14	Tin ethyl etiopurpurin dichloride	PDT	Phase II/III
15	Deuteporfin	PDT Porphyrins	Phase II
16	TLD-1433 (Code came)	PDT Ruthenium Complexes	Phase II
17	Fimaporfin/gemcitabine	PDT	Phase II
18	Exeporfinium chloride	PDT Porphyrins	Phase II
19	Photochlor	PDT	Phase II
20	Tin (IV) protoporphyrin IX dichloride	PDT Porphyrins Tin Complexes	Phase II
21	Lemuteporphin	PDT Porphyrins	Phase II
22	Lutetium texaphyrin Motexafin lutetium	PDT	Phase II
23	Redaporfin	PDT	Phase I/II
24	Fimaporfin/bleomycin	PDT	Phase I/II
25	Photocyanine	PDT Phthalocyanines Zinc Complexes	Phase I
26	Silicon phthalocyanine 4	PDT Phthalocyanines	Phase I
27	Aminolevulinic acid benzyl ester	PDT	Phase I
28	Rhodamine 123	PDT	Phase I
29	Fimaporfin	PDT	Clinical
30	Nanoshells	PTT	Clinical
31	Sinoporphyrin sodium	PDT Porphyrins Sonodynamic Therapy (SDT) Sonosensitizers	Preclinical
32	XF-70 (Code came)	PDT Porphyrins	Preclinical

## Abbreviations

PTT: Photothermal therapy
PDT: Photodynamic therapy
NIR: Near-infrared
PSs: Photosensitizers
PS: Photosensitizer
^1^O_2_: Singlet oxygen
HPD: Hematophorphyrin derivative
ROS: Reactive oxygen species
MB: Methylene blue
DPP-TPA: Pyrrolo-pyrrole-triphenyl lamine organic nanoparticles
PAI: Photoacoustic imaging
PAT: Photoacoustic tomography
GN: Gold nanorods
WP5: Column aromatic hydrocarbon
5-ALA: 5-aminolevulinic acid hydrochloride
NMRI: Nuclear magnetic resonance imaging
SPIONs: Superparamagnetic iron oxide nanoparticles
PET: Positron emission tomography
SPECT: Single-photon emission tomography
SPR: Surface plasmon resonance
18-FDG: 2-deoxy-2-[(18)F]fluoro-D-glucose
GNR: Gold nanorods
PDA NPs: Polydopamine nanoparticles
